# The Incidence and Variation of Corona Mortis in Multiracial Asian: An Insight from 82 Cadavers

**DOI:** 10.5704/MOJ.2403.004

**Published:** 2024-03

**Authors:** NA Khirul-Ashar, II Ismail, P Hussin, NM Nizlan, MH Harun, M Mawardi, R Lingam

**Affiliations:** 1 Department of Orthopaedic and Traumatology, Universiti Teknologi MARA, Sungai Buloh, Malaysia; 2 Department of Orthopaedics, Universiti Putra Malaysia, Serdang, Malaysia; 3 Department of Family Medicine, Universiti Putra Malaysia, Serdang, Malaysia; 4 Department of Orthopaedics, Hospital Serdang, Serdang, Malaysia

**Keywords:** corona mortis, vascular variant, cadaveric study, pelvis, Asian

## Abstract

**Introduction:**

Corona Mortis (CMOR) is a term used to describe an anatomical vascular variant of retropubic anastomosis located posterior to superior pubic ramus. We aim to provide sufficient data on the incidence, morphology and mean location of ‘crown of death’ in Asian population. Other objectives include to assess the relationship between CMOR incidence with gender, race and age.

**Materials and methods:**

This is a cross-sectional cadaveric study involving 164 randomly selected fresh multiracial Asian hemipelves (82 cadavers). Hemipelves were dissected to expose and evaluate the vascular elements posterior to superior pubic rami. Data were analysed using Chi-Square, t-test and with the help of IBM SPSS Statistics v26 software.

**Results:**

CMOR was found in 117 hemipelves (71.3%). No new morphological subtype was found. The mean distance of CMOR to symphysis pubis was 54.72mm (SD 9.35). Based on the results, it is evident that precaution needed to be taken at least within 55mm from symphysis pubis during any surgical intervention. The lack of statistically significant correlation between CMOR occurrence and gender, race and age suggest that the incidence of CMOR could be sporadic in manner.

**Conclusion:**

We conclude that CMOR is not just aberrant vessel as the incidence is high and this finding is comparable to other studies. The mean location of CMOR obtained in this study will guide surgeons from various disciplines in Asia to manage traumatic vascular injury and to perform a safe surgical procedure involving the pelvis area.

## Introduction

Corona Mortis (CMOR) consists of two terminologies of Latin origin, where the word ‘corona’, when used in anatomical nomenclature is to designate a crown-like eminence or encircling structure, and the word ‘mortis’ comes from the term ‘mort’ which means death^1^. The latter word attests to the significance of this feature in which since notable cause of haemorrhage from injury may lead to difficulty in achieving subsequent haemostasis. However, with the understanding of vessel course, further complication can be prevented.

CMOR is defined as an anatomical variant of retropubic anastomosis located posterior to superior pubic ramus, a communication between internal and external iliac arteries; through obturator artery or internal epigastric artery^2^. Currently, several literatures have widely accepted the definition to include the arterial and/or venous vascular communication^3,4^. It is described as an anatomical variant with variable incidence, size and location. Prevalence of these vascular connections also displays ethnic and regional differences. Previously, it was considered a rare condition however, as more case series and studies have been conducted, it became apparent that the occurrence was underestimated.

Knowledge on corona mortis is important in managing patients with pelvic injury and during surgical procedures pertaining to this vascular variant. While literatures from the Western world area readily available to serve as a guide, it would be ideal to have more applicable data from our Asian population. We anticipate that this study will act as a trailblazer in exploring the morphology and identifying mean location of CMOR among multiracial Asian hemipelves.

The main objective of this study is to determine the incidence of CMOR among multiracial Asian cadaver hemipelves. We wish to determine CMOR mean location, diameter, origin and its subtypes. Secondary objectives are to determine relationship between incidence of CMOR versus ethnicity, gender and age and its origin. We hypothesise the diameter of venous variant will be larger compared to artery. Other hypothesis include the length of CMOR is longer in females due to anthropometric differences.

## Materials and Methods

This study was a descriptive observational cross-sectional study using primary data. Data were obtained from measurements taken from cadavers which needed to undergo post-mortem autopsy in Forensic Department of our centre. Study duration was 12 months in total, starting from July 2019 until June 2020.

Our study only included adult Asian population aged more than 18 years old. We excluded decomposed cadavers, cadavers with crushed pelvic injury or in cadaver with previous scars around pelvic area that may alter its anatomy. Only fresh cadavers (i.e., within two days) were included. In this study, post-mortem was performed within 2 to 45 hours (mean time of 12.44 hours) from the pronounced time of death. Discrepancy in the timing of post-mortem was due to clearance time from Malaysian police and availability of family members to give consent. Cadavers were kept in fridge and thawed prior to post-mortem. Post-procedure, cadavers were released to family for funeral arrangement.

The study was designed to obtain a sample size within 10% of the true value at 95% confidence level with incidence of CMOR in multiracial Asian hemipelves based on the radiological study using Dual Energy CT scanner by Rahim *et al*^5^ In that study, they found 16 out of 132 hemipelves were positive Corona Mortis and thus concluded that the incidence of Corona Mortis is 0.12%.

The sample size calculation was based on a formula used to calculate approximate sample size for cross-sectional (one group) for proportion as described by Lu Ann Aday and Llewellyn J Cornelius in their book, ‘Designing and Conducting Health Surveys’^6^ using the following formula:

n=Zi−α/22P(1−P)d2

where,

Zi−α/2=z-statistic for 95% confidence interval                P=estimated proportion                d=desired precision=1.962×0.12(1−0.12)0.052

where,

Zi−α/2=1.96                P=16132=0.13                d=5%  =0.05=162 hemipelves

From the calculation, the minimal sample size needed was 162 hemipelves or equivalent to 81 cadavers.

Cadavers were selected at random. One set of 366 days from July 2019 until June 2020 was numbered 1 to 366. Next, 81 dates were chosen randomly with the help of an online application called Research Randomizer, accessible at https://www.randomizer.org/.

**Fig 1:**
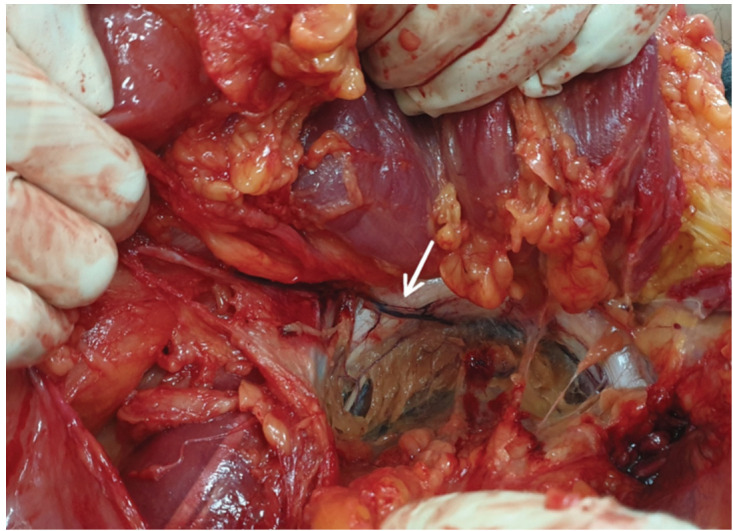
A vein (white arrow) crossing the suprapubic visible in the hemipelvis of a fresh cadaver.

Abdominal contents including bowels were carefully removed through standard “Y” shaped incision or modified “Y” shaped autopsy incision. Bladder was slowly and carefully separated from symphysis pubis to get into space of Retzius. The vascular elements posterior to the superior pubic rami were exposed and identified (Fig. 1). Origin of vessel was determined by its characteristics. Generally, arterial vessels are thicker and more reddish while venous vessels are thinner and more bluish. These vessels were traced up to external iliac vessel to confirm its origin.

The data were collected by using a proforma which includes the origin and type of the anastomosis of CMOR, the diameter of vessel, length of vessel and distance of CMOR from symphysis pubis. Measurements were made using an electronic vernier caliper in millimetre with two decimal point. All the readings were done independently by a sum of 3 readings by the same two members of the research team. Each sample’s demographic details such as cadaveric number, gender, race, age, time of death and time of post-mortem autopsy were also included in the proforma.

The independent and dependent variables were both categorical and numerical. Therefore Chi-Square, t-test and ANOVA were conducted to evaluate our research hypothesis with the help of IBM SPSS Statistics v26 software.

The study protocol was approved by Medical Research and Ethics Committee (MREC) of the National Medical Research Register (NMRR) (approval number NMRR-17-1344-34712, issued on July 11, 2017) and Institutional Review Board (IRB) (approval number HSDG/P/CRC/710/11/9(343), issued on September 11, 2017). All methods were performed in accordance with the relevant guidelines and regulations.

## Results

Based on sample size calculation, we have planned to recruit at least 81 cadavers which is equivalent to 162 hemipelves from 81 dates in a year. Among randomly chosen 81 dates, one date had two cases on the day, resulting in 82 cadavers recruited and thus resulted in 164 hemipelves being involved.

CMOR was found in 73 cadavers (89%) and absent in 9 cadavers (11%). Forty-four cadavers were found to have CMOR in bilateral hemipelves while 29 cadavers were found to have CMOR in unilateral hemipelves. From the 164 dissected hemipelves, CMOR was found in 117 (71.3%) hemipelves and absent in 47 (28.7%) hemipelves.

A total of 23 cadavers (28.0%) were Malay, 28 cadavers (34.1%) were Chinese, and 14 cadavers (17.1%) were Indian. Other races constitute 17 (20.7%) of the total samples. This comprised nine Indonesians, five Bangladeshis, and one Aborigine, one Burmese and one Thai. Sixty-six cadavers (80.5%) were males and 16 cadavers (19.5%) were females. The age range was 18 to 79 years old and the mean age was 42.48 (SD 15.55).

Out of the total 117 CMOR found in hemipelves, 57 (48.7%) were arterial in origin and 60 (51.3%) were venous in origin. In this study, no concurrent arterial and venous type of CMOR was found in ipsilateral hemipelves.

**Fig 2:**
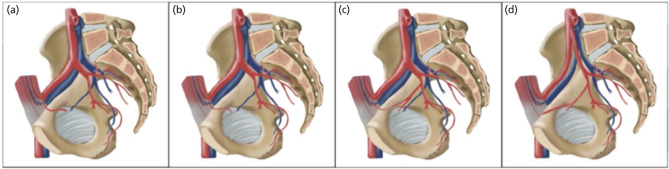
CMOR arterial subtypes. (a) - Type I.1 - obturator artery that branches from external iliac artery crosses superior pubic ramus and advances towards obturator foramen. (b) - Type I.2 - obturator artery that originates from inferior epigastric artery; which is a branch of external iliac artery, crosses superior pubic ramus and enters obturator foramen. (c) - Type I.3 - anastomotic vessel that crosses superior pubic ramus forms by obturator artery and inferior epigastric artery. (d) - Type I.4 - the pubic branch from obturator artery, does not form anastomosis but crosses over superior pubic ramus.

**Fig 3:**
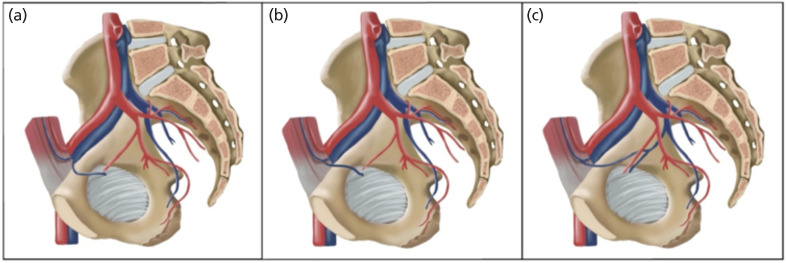
CMOR venous subtypes. (a) - Type II.1 - obturator vein crosses superior pubic ramus and directly drains into the external iliac vein. (b) - Type II.2 - obturator vein crosses superior pubic ramus and drains into inferior epigastric vein, which then later drains into external iliac vein. (c) - Type II.3 - anastomotic vessels formed by obturator vein and inferior epigastric vein crossing superior pubic ramus.

According to Rusu *et al*^2^, there are two vascular components of CMOR - arterial (I) and venous (II) in origin (Fig. 2 and 3). In our study, the most common arterial CMOR was type 1 (64.9%), followed by type 2 (26.3%), type 3 (7.0%) and type 4 (1.8%). The incidence of venous CMOR in decreasing manner were type 1 (56.6%), type 2 (36.7%) and type 3 (6.7%).

The mean diameter is 2.86mm (SD 0.73) while the mean length of CMOR is 25.86mm (SD 11.85). The mean for distance of CMOR to symphysis pubis is 54.72mm (SD 9.35). The mean diameter of arterial CMOR is 2.61mm (SD 0.62) while the mean diameter of venous CMOR is 3.09mm (SD 0.75). There is a statistically significant difference of the mean diameter between two types of CMOR origin (p<0.05). Venous CMOR was found to have a bigger diameter.

Based on Chi-Square test, there is no association between the race, gender and age in relation to the incidence of CMOR as the p value is more than 0.05. On the other hand, significant difference exists when comparing mean length between the two genders. The mean length of CMOR found in female is 19.58mm (SD 10.14) and the mean length of CMOR found in male is 28.00mm (SD 12.62).

## Discussion

CMOR was found in nearly three quarter of our measured hemipelves. Our results are comparable to previous cadaveric studies conducted^7-12^. In surgical in-vivo setting and angiography imaging, the incidence of CMOR seems to be lower (Table I)^5,13-16^. The lower incidence of CMOR in laparoscopic surgery could probably be attributed to the amount of insufflated gas in the preperitoneal space exceeding 10mmHg. In order to provide clear identification of all vessels which would decrease the potential risk of vascular injury, the proposed manoeuvre suggested by Kinaci *et al*^17^ is to decrease the pressure in the workspace to 8mmHg.

**Table I:** List of previous studies done on incidence of CMOR.

Authors	Specimen type	Sample size (n)	Incidence
Karakurt (2002)^13^	CT angiography	98	28.5%
Lau (2003)^14^	Laparoscopic	121	40.0%
Okcu (2004)^7^	Cadaveric	150	61.0%
Hong (2004)^8^	Cadaveric	50	72.0%
Pungpapong (2005)^9^	Cadaveric	66	77.2%
Namking (2006)^10^	Cadaveric	204	22.5% (artery) 70.6% (venous)
Darmanis (2007)^11^	Cadaveric	80	83.0%
Smith (2009)^15^	CT venous	100	29.0%
Rahim (2014)^5^	CT angiography	132	12.0%
Pellegrino (2014)^16^	Laparoscopic	50	52.0%
Ruangwannasak (2019)^12^	Cadaveric	40	60.0%

The incidence of CMOR according to its origin is similar in arterial and venous variants. No new subtype of CMOR variant found in this study.

The mean location of CMOR found in this study in comparison to previous studies in this region is within the same range (52 to 64mm) (Table II)^5,7-10,12^. However, Western studies conducted in United Kingdom and United States show a wider range of up to 72mm (Table III)^11,15^. This could be attributed to the larger build of Westerners, in comparison to Asians.

**Table II:** List of previous studies done on incidence, mean location and mean size of CMOR among Asian population.

Authors	Specimen type	Incidence	Mean location to symphysis pubis (mm)	Mean diameter (mm)	Country
Hong (2004)^8^	Cadaveric	72.0%	52.0	2.6	China
Okcu (2004)^7^	Cadaveric	61%	64.0 (arterial) 56.0 (venous)	N/A	Turkey
Pungpapong (2005)^9^	Cadaveric	77.0%	52.8	N/A	Thailand
Namking (2006)^10^	Cadaveric	22.5% (artery) 70.6% (venous)	N/A	N/A	Thailand
Rahim (2014)^5^	CT angiography	12.0%	48.0	1.2 - 2.8 (1.8)	Malaysia
Ruangwannasak (2019)^12^	Cadaveric	60.0%	22.1 - 45.0	N/A	Thailand

**Table III:** List of global previous studies done on mean location of CMOR.

Authors	Sample size (n)	Mean location to symphysis pubis (mm)	Country
Okcu (2004)^7^	150	64.0 (arterial) 56.0 (venous)	Turkey
Hong (2004)^8^	50	52.0	China
Pungpapong (2005)^9^	66	52.8	Thailand
Darmanis (2007)^11^	80	71.0 (arterial) 65.0 (venous)	United Kingdom
Smith (2009)^15^	100	56.0 (range: 41.0-72.0)	United States
Rahim (2014)^5^	132	48.0	Malaysia
Ruangwannasak (2019)^12^	40	22.1 - 45.0	Thailand

The mean diameter of CMOR is 2.86mm (SD 0.73) and is comparable to other studies done in this region (Table II)^5,8^. There is a significant difference found in mean diameter of CMOR where the venous CMOR of 3.09mm (SD 0.75) was found to have a bigger diameter as compared to the arterial CMOR of 2.61mm (SD 0.62). This corresponds to normal anatomy of vessel itself whereby diameter of veins are larger than the arteries despite the arteries having thicker wall. The mean length of CMOR is 25.86mm (SD 11.85).

Based on the statistical analysis, there was no correlation between the race, gender and age in relation to the incidence of CMOR and hence might suggest that the occurrence of CMOR is probably in sporadic manner. This finding is supported by previous study by Rahim *et al*^5^, that showed insignificant difference between race, gender and age.

Females have wider pelves and we postulate the length of CMOR would be longer in comparison to males, but our result showed otherwise. Hence, we wish to outline a few limitations that we have identified in our study. This study has the typical limitations all examinations in cadavers have in contrast to living samples such as stretching of the vessels during exploration of hemipelvis that might alter its size and length. Apart from that, measurements could also be inaccurate due to lack of blood circulation. It is still uncertain how big is the size of injured CMOR vessels which could cause significant morbidity and mortality in our clinical settings.

Other than that, is the relatively small sample size involved. With a larger sample size, the significance of the study would have been magnified and it would reflect a more accurate incidence of CMOR incidence in multiracial Asian hemipelves.

Another limitation is the unbalanced ethnicity of the samples which comprised of 28.0% Malays, 34.1% Chinese, and 17.1% Indians. Other races constitute 20.7% of samples which include 9 Indonesians, 5 Bangladeshis, 1 Aborigine, 1 Burmese and 1 Thai. While this is a stepping-stone for a more comprehensive study to reflect multiracial Asian population in the future, until then conclusions obtained from this study can only be applied for certain groups of patients in our clinical practice.

It is important for orthopaedic and vascular surgeons together with interventional radiologists to be aware of the probability of injury to this vessel following a traumatic pelvic injury. Thus, reducing morbidity rate and to prevent ‘early deaths’ that occur in the first few hours following major trauma. Apart from that, it is important for the surgeons to be familiar with the course and location of this vessel in surgical planning so that life-threatening injury from massive bleeding can be avoided. Previous studies confirmed the significance of CMOR intraoperatively whereby surgeon may accidentally injure this vessel thus lead to a catastrophic event. This unexpected presence of CMOR can become a matter of great concern among surgeons (including general surgeon, urologist, gynaecologist and orthopaedic surgeon) who perform any surgical procedure in the pelvic region. Despite the fear of excessive bleeding, surgical approach should not be altered but extra caution should be exercised instead.

## Conclusion

We conclude that CMOR is not just aberrant vessel as the incidence is high and this finding is comparable to other studies. The mean diameter is 2.86mm and bigger diameter found in venous type. In accordance with the data obtained in this study, precaution should be taken 55mm from the symphysis pubis during procedures or surgeries in this region for Asian hemipelves. Its occurrence seems to be sporadic in nature.
